# Effects of Supplementation of the Synbiotic Ecologic^®^ 825/FOS P6 on Intestinal Barrier Function in Healthy Humans: A Randomized Controlled Trial

**DOI:** 10.1371/journal.pone.0167775

**Published:** 2016-12-09

**Authors:** E. Wilms, J. Gerritsen, H. Smidt, I. Besseling-van der Vaart, G. T. Rijkers, A. R. Garcia Fuentes, A. A. M. Masclee, F. J. Troost

**Affiliations:** 1 Division of Gastroenterology-Hepatology, Department of Internal Medicine, NUTRIM School of Nutrition and Translational Research in Metabolism, Maastricht University Medical Center+, Maastricht, the Netherlands; 2 Laboratory of Microbiology, Wageningen University, Wageningen, the Netherlands; 3 Winclove Probiotics BV, Amsterdam, the Netherlands; 4 Science Department, University College Roosevelt, Middelburg, the Netherlands; 5 Department of Medical Microbiology and Immunology, St. Antonius Hospital, Nieuwegein, the Netherlands; University Hospital Llandough, UNITED KINGDOM

## Abstract

**Background and Aims:**

Probiotics, prebiotics and synbiotics have been suggested as dietary strategies to improve intestinal barrier function. This study aimed to assess the effect of two weeks synbiotic supplementation on intestinal permeability under basal and stressed conditions. Secondary aims were the assessment of two weeks synbiotic supplementation on systemic immune function and gastrointestinal symptoms including defecation pattern.

**Design:**

Twenty healthy adults completed a double-blind, controlled, randomized, parallel design study.

**Intervention:**

Groups either received synbiotic (1.5 × 10^10^ CFU Ecologic^®^ 825 + 10 g fructo-oligosaccharides (FOS P6) per day) or control supplements for two weeks.

**Outcomes:**

Intestinal segment specific permeability was assessed non-invasively by oral administration of multiple sugar probes and, subsequently, assessing the excretion of these probes in urine. This test was conducted at baseline and at the end of intervention, in the absence and in the presence of an indomethacin challenge. Indomethacin was applied to induce a compromised gut state. Plasma zonulin, cytokines and chemokines were measured at baseline and at the end of intervention. Gastrointestinal symptoms and stool frequency were recorded at baseline and daily during intervention.

**Results:**

**S**ignificantly more male subjects were in the synbiotic group compared to the control group (*P* = 0.025). Indomethacin significantly increased urinary lactulose/rhamnose ratio versus without indomethacin, both in the control group (*P* = 0.005) and in the synbiotic group (*P* = 0.017). Urinary sugar recoveries and ratios, plasma levels of zonulin, cytokines and chemokines, and gastrointestinal symptom scores were not significantly different after control or synbiotic intervention. Stool frequency within the synbiotic group was significantly increased during synbiotic intervention compared to baseline (*P* = 0.039) and higher compared to control intervention (*P* = 0.045).

**Conclusion:**

Two weeks Ecologic^®^ 825/FOS P6 supplementation increased stool frequency, but did not affect intestinal permeability neither under basal nor under indomethacin-induced stressed conditions, immune function or gastrointestinal symptoms in healthy adults.

## Introduction

Epithelium integrity of the gastrointestinal tract is pivotal for maintainance of the intestinal barrier. Apart from epithelial cells, the activation state of the immune system, intestinal microbiota and their metabolism, mucus production, secretion of antimicrobial peptides, tight junction proteins and the enteric nervous system all contribute to maintaining the intestinal barrier. Patients with intestinal diseases such as inflammatory bowel disease (IBD) [1,2] or celiac disease [3,4] show increased intestinal permeability. It is not clear whether increased intestinal permeability is a causal factor or a consequence of intestinal disorders, however, in a subset of relatives of symptom-free IBD patients intestinal permeability was found to be increased [5]. These subjects have a significantly inceased risk to develop IBD, suggesting that changes in intestinal permeability precede the development of intestinal disease. Reinforcement of (disturbed) intestinal barrier may thus become an important target in prevention and treatment of intestinal disorders [6].

Prebiotics and probiotics have been proposed as promising interventions to improve intestinal barrier function. Indeed, in several studies the effects of probiotics and prebiotics on intestinal permeability have been investigated in healthy volunteers. While some human intervention studies found evidence for improvement in intestinal permeability after either probiotic [7,8], prebiotic [9], or synbiotic [10] consumption, others did not observe any change in permeability with a prebiotic product [11].

Recently, *in vitro*, *ex vivo* and animal studies have shown positive effects of the multispecies probiotic mixture Ecologic^®^ 825 on intestinal barrier function [12–14]. Our aim was to evaluate the effect of this mixture Ecologic^®^ 825 on intestinal barrier function in healthy volunteers. In addition we chose to fortify the multispecies probiotic mixture with fructo-oligosaccharides (FOS P6) to stimulate selective growth and activity of the probiotic strains. We hypothesized that a two-week synbiotic supplementation will decrease intestinal permeability in healthy adults, both under basal conditions and under conditions of mucosal stress, induced by administering the non-steroidal anti-inflammatory drug indomethacin. The primary aim was to assess the effect of two weeks synbiotic supplementation on intestinal permeability under basal and stressed conditions. Secondary aims were to evaluate the effect of two weeks synbiotic supplementation on systemic immune function, gastrointestinal symptoms and stool frequency.

## Methods

This last version of the study protocol was approved by the Medical Ethics Committee of the Maastricht University Medical Center + at 13 November 2013, and performed in accordance with the Declaration of Helsinki (latest amendment by the World Medical Association in 2013) and Dutch Regulations on Medical Research involving Human Subjects (WMO, 1998). The study was performed at the Maastricht University Medical Center + from 20 November 2013 to 28 May 2014. This study was part of a larger study which has been registered in the US National Library of Medicine (http://www.clinicaltrials.gov, ID NCT02018900) at 9 December 2013, accidentally after enrolment of the first participants. The authors confirm that all ongoing and related trials for this intervention have been registered. The study protocol and CONSORT checklist are available as supporting information files (S1 File and S2 File). The study protocol included a detailed analysis of microbiota composition and functionality along the gastrointestinal tract by sampling content from the duodenum, jejunum, ileum and feces. Those data will be published in a separate manuscript. All subjects gave written informed consent before screening.

### Subjects

Healthy men and women were recruited by local advertisements. Inclusion criteria included age between 18 and 65 years, and body mass index (BMI) between 20 and 30 kg/m^2^. Exclusion criteria included gastrointestinal symptoms, history of any chronic disorder, allergy, major surgery, self-reported human immunodeficiency virus, excessive alcohol consumption (> 20 alcohol units per week), smoking, pregnancy, lactation, use of any medication or vitamin supplements 14 days prior to testing, use of antibiotics 90 days prior to testing, blood donation three months prior to testing, use of pro- or prebiotics 180 days prior testing, and a history of side effects towards pro- or prebiotic supplements.

### Sample size calculation

**Fig 1:**
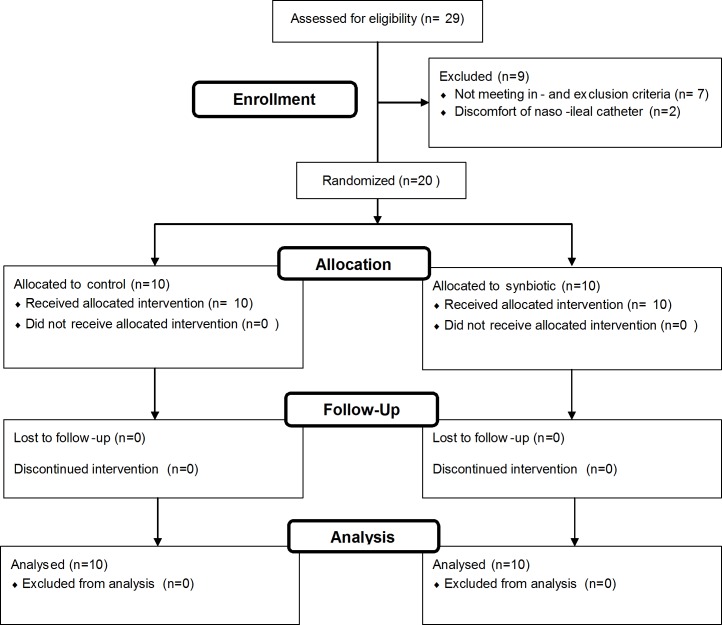
Flow diagram of the study.

The sample size calculation was based on the difference in urinary lactulose recovery between indomethacin ingestion and placebo ingestion as reported by van Wijck *et al*. [15]. We assumed a difference between treatments of 3.04 μmol (20%), standard deviation of 2.10 μmol, an alpha of 0.05, and a power of 0.80. Based on this calculation, 9 participants per group were needed to complete the study to reach sufficient statistical power. We included 10 participants per group because of the estimated dropout rate of 10% (Fig 1).

### Study design

**Fig 2:**
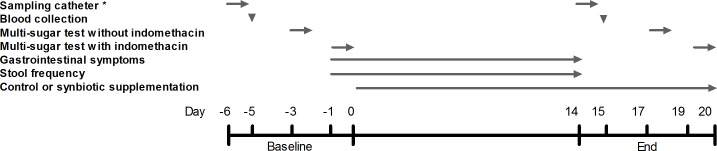
Timeline of the intervention period. Sampling catheter, blood collection, multi-sugar tests, gastrointestinal symptoms, stool frequency, and control or synbiotic supplementation were completed at the days as indicated by arrows. * Data not in present manuscript.

The study was a double-blind, randomized, controlled, parallel design study. Participants were randomly and equally assigned to the control or synbiotic group. The randomization list was generated by using a computerized procedure. All participants and investigators remained blinded to treatment until all analyses were completed. Subjects in the synbiotic group received synbiotic supplements that were composed of a multispecies probiotic mixture (Ecologic^®^ 825, 6 g/day, 1.5 *10^10^ colony-forming units/day); Winclove Probiotics BV, Amsterdam, the Netherlands) comprising *Bifidobacterium bifidum* (W23), *B*. *lactis* (W51), *B*. *lactis* (W52), *Lactobacillus acidophilus* (W22), *L*. *casei* (W56), *L*. *paracasei* (W20), *L*. *plantarum* (W62), *L*. *salivarius* (W24) and *Lactococcus lactis* (W19) combined with fructooligosaccharides (FOS P6, degree of polymerization between 3 and 5, 10 g/day; Winclove Probiotics BV, Amsterdam, the Netherlands). Subjects in the control group received the same carrier material as the multispecies probiotic mixture (6 g/day; Winclove Probiotics BV, Amsterdam, the Netherlands), but without probiotic strains, combined with maltodextrin (10 g/day; Winclove Probiotics BV, Amsterdam, the Netherlands) instead of FOS. Carrier material comprised maize starch, maltodextrins, a mineral mix, inulin and FOS (P6; inulin and FOS comprised maximum 15% of the total carrier material). Synbiotic and control mixtures had an identical appearance and were supplied in duo sachets. Subjects ingested the mixtures every morning and evening at the same time, for two weeks. The total sachet content was dissolved in 200 ml lukewarm water, left for 10 min to mix and dissolve, stirred and subsequently ingested. The time of consumption was recorded in a diary. All empty and remaining duo sachets were returned to the investigator. At day -6 and day 14 a multi-lumen customized sampling catheter (Mui Scientific, Mississauga, Ontario, Canada) was placed in the small intestine for analysis of small intestinal microbiota composition and functionality (data will be published in a separate manuscript). To determine plasma zonulin, cytokine- and chemokine concentrations, blood samples were taken at day -5 and day 15. Intestinal permeability was examined by multi-sugar tests without indomethacin challenge at day -3 and day 17 and with indomethacin challenge at day -1 and day 19. Gastrointestinal symptom scores and stool frequency were recorded once daily at baseline and during 14 days of supplementation. (Fig 2)

### Intestinal permeability

Permeability of different segments of the gastrointestinal tract was assessed non-invasively by multi-sugar tests as validated by van Wijck *et al*. [15,16]. One day prior to testing, and during all test days, excessive physical exercise and consumption of alcohol were not allowed. Water-soluble, non-degradable sugar probes were ingested after fasting overnight. Subjects ingested 1 g sucrose (Van Gilse, Dinteloord, the Netherlands), 1 g lactulose (Centrafarm Services, Etten-Leur, the Netherlands), 0.5 g L-rhamnose (Danisco sweeteners, Thomson, IL, USA), 1 g sucralose (Tate and Lyle Ingredients Americas, Decatur, IL, USA) and 1 g erythritol (Now Foods, Bloomindale, IL, USA). Subjects collected 24 hours (h) urine in two separate containers; 0–5 h and 5–24 h after sugar ingestion. Subjects were not allowed to consume food or drinks, except for water *ad libitum*, during the first 5 h of urine collection. After these 5 h, subjects were allowed to eat and drink as preferred, with the exception of sucralose containing foods. Indomethacin was ingested to induce standardized, reversible damage to the healthy small intestine [15]. Exactly nine hours and one hour prior to the intake of the multi-sugar drink, subjects ingested 75 mg and 50 mg of indomethacin Retard (Mylan, Bunschoten, the Netherlands), respectively. After urine collection, urine was handed in, volumes of urine fractions were determined and urine aliquots were frozen at -80°C until analysis. Sugar probes were analyzed by isocratic ion-exchange High Performance Liquid Chromatography with mass spectrometry as described previously [15,16]. Gastroduodenal permeability was determined by sucrose excretion in 0–5 h urine, whereas small intestinal permeability was measured by lactulose excretion and the lactulose to rhamnose (L/R) ratio in 0–5 h urine. Sucralose excretion as well as the sucralose to erythritol (S/E) ratio in 5–24 h urine were used as indicators for colonic permeability. Rhamnose excretion in 0–5 h urine and erythritol excretion in 5–24 h urine were measured and reported, albeit these are no parameters of intestinal permeability. Further, participants fasted for at least ten hours before blood sampling. Blood was collected in BD Vacutainer^®^ K_2_EDTA tubes (BD, Breda, The Netherlands), and centrifuged at 3000 × g for 10 min at 4°C. Plasma was stored at -80°C until analysis. As biomarker of intestinal barrier disruption, active uncleaved zonulin was measured in blood plasma by using a standard Zonulin Enzyme-Linked ImmunoSorbent Assay Kit (K5601, Immundiagnostik AG, Bensheim, Germany) and expressed as ng/ml blood plasma.

### Immune function

Plasma levels of Tumor necrosis factor-alpha (TNF-α), Interleukin (IL)-1b, IL-6, IL-8, IL-17, Monocyte chemoattractant protein-1 (MCP-1) and macrophage inflammatory protein-1 alpha (MIP-1α) were measured by using the Bio-Plex Pro^TM^ Assay (Bio-Rad Laboratories, Inc., Hercules, CA, USA) and expressed as pg/ml blood plasma. IL-17 and MIP-1α were excluded from statistical analyses because values were under the detection limit of the assay.

### Gastrointestinal symptoms and stool frequency

At baseline and during the 14 days supplementation period participants completed a ‘symptoms diary’ at the end of each day. This non-validated questionnaire has been described before by Salden *et al*. [17]. Feelings of abdominal discomfort, abdominal pain, abdominal distension, constipation, diarrhea, flatulence, eructation, nausea and total discomfort were assessed by scores from 1 (no symptoms) to 5 (pronounced symptoms). In addition, stool frequency was assessed as number of bowel movements per day.

### Statistical analyses

The primary outcome of the study was the effect of two weeks synbiotic supplementation on intestinal permeability under basal and stressed conditions. Secondary outcomes were the effects of two weeks synbiotic supplementation on systemic immune function and on gastrointestinal symptoms including stool frequency. Statistical analyses were performed using IBM SPSS Statistics Version 21 (IBM Corporation, Armonk, NY, USA). A Shapiro-Wilk test was performed to test for normality. Gender was compared between intervention groups by a Chi-square test. Age, BMI, parameters of intestinal permeability, zonulin concentration, cytokine- and chemokine concentrations, and average symptom scores were compared non-parametrically. Mann-Whitney U tests and a Wilcoxon signed-rank tests were used to compare data between and within groups, respectively. Stool frequency was normally distributed. An independent samples t-test and a paired samples t-test were performed to compare between and within group data, respectively. For all analyses a two-sided test was performed and *P*<0.05 was considered statistically significant.

## Results

### Subjects

As shown in the flow diagram (Fig 1), a total of 29 volunteers were recruited. Seven volunteers did not meet the in- and exclusion criteria. Two participants terminated the study before allocation of the intervention, due to discomfort of the naso-ileal catheter which was placed to sample small intestinal contents. Therefore, 20 participants completed the entire study protocol and were included in the analyses. In the control group (n = 10) 30% were males, with a median age of 21.7 [20.0–24.0] years and median BMI of 24.1 [22.9–24.9] kg/m^2^. In the synbiotic group (n = 10) 80% were males, with a median age of 19.7 [19.1–21.8] years and median BMI of 22.9 [21.7–24.1] kg/m^2^. (Table 1)

**Table 1:** Baseline characteristics of the control group (n = 10) and synbiotic group (n = 10). Values are presented as median and IQR (25-75th interquartile range). Gender was compared between groups with the use of a Chi-square test. Age and BMI were compared between groups with the use of a Mann-Whitney U test. BMI, Body Mass Index.

	Control (n = 10)	Synbiotic (n = 10)	*P*-value
Gender (male: female)	3: 7	8: 2	0.025
Age (yrs)	21.7 [20.0–24.0]	19.7 [19.1–21.8]	0.082
BMI (kg/m2)	24.1 [22.9–24.9]	22.9 [21.7–24.1]	0.226

### Gastrointestinal permeability

Effect of indomethacin: Urinary sugar excretions and ratios at baseline are given in Table 2. Data of urinary sugar excretion after indomethacin challenge were compared to data of urinary sugar excretion without indomethacin challenge. In the synbiotic group, indomethacin significantly decreased urinary rhamnose excretion and significantly increased urinary L/R ratio. In the control group, indomethacin significantly increased urinary sucrose excretion, urinary lactulose excretion and urinary L/R ratio, pointing to increased gastroduodenal and small intestinal permeability. Urinary sucralose excretion, urinary erythritol excretion and urinary S/E ratio were not affected by indomethacin.

**Table 2:** Urinary sugar excretions (μmol) and ratios of the control group (n = 10) and synbiotic group (n = 10) at baseline without and with indomethacin challenge. Values are presented as median and IQR (25-75th interquartile range). Urinary sugar excretions and ratios without indomethacin vs. with indomethacin were compared with the use of a Wilcoxon signed-rank test; L/R, lactulose/rhamnose; S/E, sucralose/erythritol.

Urinary sugar excretion	Control			Synbiotic		
	Without indomethacin	With indomethacin	*P-*value	Without indomethacin	With indomethacin	*P-*value
0–5 h sucrose	6.84 [5.65–9.12]	9.67 [8.50–18.94]	0.022	7.24 [6.11–10.89]	13.28 [6.03–19.50]	0.333
0–5 h lactulose	6.19[5.14–7.24]	11.73[9.30–16.27]	0.005	14.08 [6.30–23.03]	18.92 [11.45–31.76]	0.139
0–5 h rhamnose	287 [216–336]	252 [163–327]	0.285	395 [280–603]	213 [241–358]	0.037
0–5 h L/R ratio	0.023[0.020–0.026]	0.061[0.042–0.074]	0.005	0.032 [0.022–0.043]	0.064 [0.046–0.106]	0.017
5–24 h sucralose	39.48 [31.54–93.49]	47.00[37.89–63.15]	0.799	59.29 [49.75–71.59]	54.29 [43.16–95.52]	0.445
5–24 h erythritol	3291 [2490–3523]	2731[2164–3073]	0.093	3163 [2483–3460]	2845 [1700–3469]	0.285
5–24 h S/E ratio	0.016 [0.011–0.023]	0.021 [0.012–0.024]	0.646	0.019 [0.014–0.023]	0.023 [0.014–0.047]	0.241

Effect of synbiotic supplementation: Within the synbiotic group, no significant differences were found when comparing values observed at baseline and after intervention in urinary sucrose excretion, urinary lactulose excretion, urinary rhamnose excretion, urinary L/R ratio, urinary sucralose excretion, urinary erythritol excretion or urinary S/E ratio, neither without nor with indomethacin challenge (Table 3). Besides the significantly higher urinary rhamnose excretion after intervention with indomethacin challenge, no significant differences were observed in the control group with respect to urinary sugar excretions and ratios (Table 4). Also, no significant differences were observed between the synbiotic and control group after the two-week supplementation period in urinary sucrose excretion, urinary lactulose excretion, urinary rhamnose excretion, urinary L/R ratio, urinary sucralose excretion, urinary erythritol excretion, or urinary S/E ratio. Overall, synbiotic supplementation did not influence gastroduodenal, small intestinal or colonic permeability.

**Table 3:** Urinary sugar excretions (μmol) and ratios of the synbiotic group (n = 10) at baseline and after two weeks synbiotic supplementation, without and with indomethacin challenge. Values are presented as median and IQR (25-75^th^ interquartile range). Urinary sugar excretions and ratios at baseline vs. end were compared with the use of a Wilcoxon signed-rank test. L/R, lactulose/rhamnose; S/E, sucralose/erythritol.

Urinary sugar excretion	Without indomethacin			With indomethacin		
	Baseline	End	*P*-value	Baseline	End	*P*-value
0–5 h sucrose	7.24 [6.11–10.89]	10.72 [7.02–19.53]	0.059	13.28 [6.03–19.50]	13.71 [9.93–21.06]	0.959
0–5 h lactulose	14.08 [6.30–23.03]	9.34 [6.92–18.68]	0.575	18.92 [11.45–31.76]	17.91 [9.17–24.97]	0.721
0–5 h rhamnose	395 [280–603]	383 [280–436]	0.139	213 [241–358]	360 [251–414]	0.799
0–5 h L/R ratio	0.032 [0.022–0.043]	0.031 [0.024–0.044]	0.878	0.064 [0.046–0.106]	0.055 [0.037–0.072]	0.203
5–24 h sucralose	59.29 [49.75–71.59]	40.10 [33.52–74.44]	0.285	54.29 [43.16–95.52]	51.95 [40.83–64.85]	0.646
5–24 h erythritol	3163 [2483–3460]	2864 [2604–3176]	0.333	2845 [1700–3469]	3316 [2276–3602]	0.169
5–24 h S/E ratio	0.019 [0.014–0.023]	0.014 [0.013–0.026]	0.508	0.023 [0.014–0.047]	0.016 [0.015–0.022]	0.169

**Table 4:** Urinary sugar excretions (μmol) and ratios of the control group (n = 10) at baseline and after two weeks intervention, without and with indomethacin challenge. Values are presented as median and IQR (25-75^th^ interquartile range). Urinary sugar excretions and ratios at baseline vs. end were compared with the use of a Wilcoxon signed-rank test. L/R, lactulose/rhamnose; S/E, sucralose/erythritol.

Urinary sugar excretion	Without indomethacin			With indomethacin		
	Baseline	End	*P-*value	Baseline	End	*P-*value
0–5 h sucrose	6.84 [5.65–9.12]	8.69[6.76–10.94]	0.169	9.67 [8.50–18.94]	9.82[6.89–18.70]	0.646
0–5 h lactulose	6.19[5.14–7.24]	9.56 [6.59–13.29]	0.139	11.73[9.30–16.27]	18.06 [10.77–25.39]	0.203
0–5 h rhamnose	287 [216–336]	341 [269–474]	0.285	252 [163–327]	312 [260–464]	0.037
0–5 h L/R ratio	0.023[0.020–0.026]	0.025 [0.021–0.041]	0.093	0.061[0.042–0.074]	0.055 [0.041–0.071]	0.799
5–24 h sucralose	39.48 [31.54–93.49]	44.43[31.41–58.27]	0.333	47.00[37.89–63.15]	53.51[41.63–63.76]	0.386
5–24 h erythritol	3291 [2490–3523]	2565 [2035–2865]	0.059	2731[2164–3073]	3052 [2386–3395]	0.285
5–24 h S/E ratio	0.016 [0.011–0.023]	0.021 [0.013–0.023]	0.203	0.021 [0.012–0.024]	0.018[0.014–0.024]	0.959

### Zonulin

Within the synbiotic group, plasma zonulin was 14.3 [12.6–17.1] ng/ml at baseline and 13.2 [10.4–17.2] ng/ml at the end of intervention (*P* = 0.721). Plasma zonulin was 14.8 [11.6–16.3] ng/ml at baseline and 14.7 [12.1–15.6] ng/ml at the end intervention in the control group (*P* = 0.959). Moreover, at the end of intervention, plasma zonulin concentrations were not significantly different between the control group and synbiotic group (*P* = 0.650).

### Immune function

To study the effect of synbiotic supplementation on immune modulation, plasma cytokines and chemokines were determined before and after the intervention. TNF-α, IL-1β, IL-6, IL-8 and MCP-1 were not significantly different between the control and synbiotic group at baseline (all *P*>0.174). After two weeks of supplementation, neither plasma TNF-α, IL-1β, IL-6, IL-8 nor MCP-1 differed between baseline versus end in the synbiotic orcontrol group (Table 5).

**Table 5:** Plasma cytokines and chemokines of the control group (n = 10) and synbiotic group (n = 10) at baseline and after two weeks. Values are presented as median and IQR (25-75th interquartile range). Plasma cytokines and chemokines at baseline vs. end were compared with the use of aWilcoxon signed-rank test. TNF-α, Tumor necrosis factor-alpha; IL, Interleukin; MCP-1, Monocyte chemoattractant protein-1.

	Control			Synbiotic		
	Baseline	End	*P-*value	Baseline	End	*P-*value
TNF-α (pg/ml)	1.75[1.17–8.08]	1.78 [1.11–6.10]	0.374	3.55 [1.11–6.95]	3.36 [2.18–5.70]	0.799
IL-1β (pg/ml)	0.08[0.05–0.11]	0.05 [0.03–0.08]	0.112	0.10 [0.05–0.15]	0.13 [0.09–0.17]	0.540
IL-6 (pg/ml)	0.25 [0.11–0.39]	0.22 [0.01–0.50]	0.249	0.50 [0.22–1.45]	0.31 [0.25–0.60]	0.208
IL-8 (pg/ml)	0.95 [0.72–1.40]	0.94 [0.69–1.53]	0.878	1.22 [0.93–1.69]	1.39 [1.00–1.91]	0.878
MCP-1 (pg/ml)	18.79 [12.60–29.40]	19.14 [14.57–27.31]	0.878	25.96 [18.87–31.23]	25.80 [22.66–28.73]	0.333

### Gastrointestinal symptoms and stool frequency

**Fig 3:**
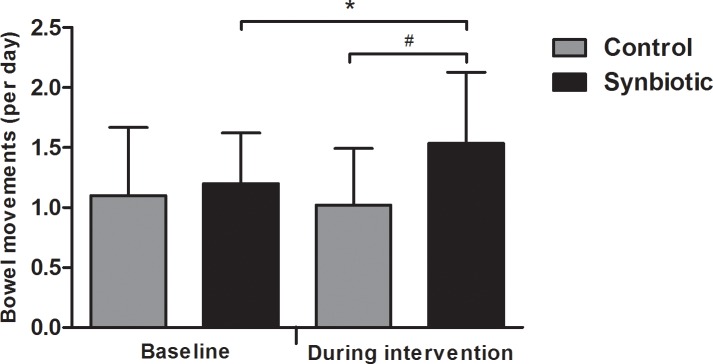
Stool frequency indicated as bowel movements per day (mean ± standard deviation) at baseline and during synbiotic (n = 10) or control (n = 10) supplementation. * Significantly increased at *P* = 0.045 based on paired samples t-test. ^#^ Significantly higher at *P =* 0.039 based on independent samples t-test.

At baseline and during the intervention period average gastrointestinal symptom scores were not significantly different between control and synbiotic supplementation (Table 6). Stool frequency did not significantly differ between the control group and synbiotic group at baseline (*P* = 0.177). Stool frequency within the synbiotic group was 1.54 ± 0.59 bowel movements per day during the intervention, which was a significant increase compared to 1.00 ± 0.47 bowel movements at baseline (Fig 3), and significantly higher compared to 1.02 ± 0.47 bowel movements per day the intervention in the control group(Fig 3).

**Table 6:** Gastrointestinal symptom scores at baseline and during control (n = 10) and synbiotic (n = 10) supplementation. Symptoms are scored on a five-point Likert scale. Values are presented as median and IQR (25-75th interquartile range). Average gastrointestinal symptom scores control vs. synbiotic were compared with the use of a Mann-Whitney U test.

	Baseline			Average during supplementation		
	Control	Synbiotic	*P-*value	Control	Synbiotic	*P-*value
Abdominal discomfort	1.00 [1.00–2.00]	1.00 [1.00–2.00]	0.687	1.39 [1.00–1.80]	1.25 [1.00–1.77]	0.670
Abdominal pain	1.00 [1.00–1.25]	1.00 [1.00–2.00]	0.726	1.18 [1.00–1.29]	1.14 [1.00–1.39]	0.908
Abdominal distension	1.00 [1.00–1.25]	1.00 [1.00–1.25]	0.914	1.18 [1.00–1.68]	1.14 [1.00–2.00]	0.938
Constipation	1.00 [1.00–1.25]	1.00 [1.00–1.00]	0.146	1.00 [1.00–1.16]	1.07 [1.00–1.46]	0.361
Diarrhea	1.00 [1.00–1.00]	1.00 [1.00–1.00]	0.317	1.14 [1.05–1.23]	1.07 [1.00–1.23]	0.461
Flatulence	1.00 [1.00–2.00]	1.00 [1.00–1.25]	0.726	1.29 [1.07–2.14]	1.50 [1.14–2.14]	0.492
Eructation	1.00 [1.00–1.25]	1.00 [1.00–2.00]	0.451	1.00 [1.00–1.14]	1.18 [1.00–1.39]	0.225
Nausea	1.00 [1.00–1.00]	1.00 [1.00–1.00]	0.317	1.00 [1.00–1.02]	1.07 [1.00–1.32]	0.084
Total discomfort	1.00 [1.00–2.00]	1.00 [1.00–2.00]	0.888	1.25 [1.00–1.71]	1.36 [1.00–2.00]	0.938

## Discussion

This study showed that supplementation with the synbiotic mixture Ecologic^®^ 825/FOS P6 did not affect intestinal permeability neither without nor with indomethacine challenge. In line with these findings, no effect of the synbiotic intervention on plasma levels of zonulin and proinflammatory cytokines and chemokines were observed. Administration of indomethacin resulted in an increase in gastroduodenal and small intestinal permeability, confirming previous observations that this serves as a reproducible model for a compromised gut [15]. As expected, colonic permeability was not affected by indomethacin administration. In contrast to our hypothesis, two weeks synbiotic supplementation did not prevent or reduce gastroduodenal and small intestinal permeability, even under compromised conditions.

Indomethacin is known to inhibit cyclooxygenase (COX)-1 and COX-2 activity in the stomach and small intestine and subsequently can cause direct damage to the enterocyte by disruption of the mitochondrial process through uncoupling of oxidative phosphorylation, leading to reduced intracellular adenosine triphosphate levels [18,19]. Reductions in adenosine triphosphate-dependent actin organisation and myosin-dependent contractility will lead to an impaired tight junction complex integrity [19]. An *in vitro* study showed that *L*. *rhamnosus* GG induced COX-2 expression in T84 cells, up to 48 h from the start of incubation [20]. Moreover, a human intervention study in healthy volunteers has shown that intake of *L*. *rhamnosus* GG significantly reduced the indomethacin-induced alteration in gastric permeability as determined by the urinary sucrose excretion, but not intestinal permeability as determined by urinary lactulose/mannitol ratio [8]. Thus, probiotics exert strain-specific and strain-dependent effects. *L*. *rhamnosus* GG was not included in the synbiotic mixture used in the present study. Ecologic^®^ 825 contains probiotic strains belonging to *B*. *bifidum*, *B*. *lactis*, *L*. *acidophilus*, *L*. *casei*, *L*. *paracasei*, *L*. *plantarum*, *L*. *salivarius* and *Lactococcus lactis*. Several *in vitro* studies showed that single strains of *B*. *bifidum* [21], *L*. *casei* [22,23] and *L*. *plantarum* [24] were able to increase transepithelial electrical resistance in intestinal epithelial cells. Although Ecologic^®^ 825 combined with FOS P6 did not reinforce intestinal barrier function in healthy adults in this study, a previous study has shown positive effects of eight weeks Ecologic^®^ 825 supplementation on intestinal barrier function in IBD patients with active pouchitis, which also received antibiotic treatment before the start of the probiotic supplementation [12]. In an Ussing chamber experiment using human ileum mucosa samples from pouchitis patients with an ileoanal pouch, the transmucosal passage of *Escherichia coli* K12 and the permeability to horseradish peroxidase were lower after probiotic supplementation, when compared to intestinal mucosa after antibiotic treatment and when compared to intestinal mucosa of healthy individuals. Therefore, it was concluded that Ecologic^®^ 825 restored the mucosal barrier in patients suffering from active pouchitis [12]. It should be noticed that IBD patients have a disturbed barrier function, whereas here we investigated healthy volunteers in whom the barrier is considered to be intact although we attempted to mimic the compromised state with the indomathic stressor protocol. Further, Ecologic^®^ 825 has been shown to exert protective effects on the colonic mucosal barrier in rat models of chronic stress. It has been suggested that these effects are modulated by a mast cell dependent pathway [13]. Ecologic^®^ 825 has been suggested to be able to modulate mast cells [14]. Mast cell function or mast cell markers or metabolites have unfortunately not been evaluated in our study.

In our study, Ecologic^®^ 825/FOS P6 supplementation did not affect plasma zonulin concentrations. Circulating zonulin is a marker of intestinal permeability, and levels are higher in cases of increased intestinal permeability [25,26]. Data on effects of probiotics or prebiotics on circulating zonulin levels are limited. In one randomized crossover study serum zonulin was found to be decreased by five weeks inulin-enriched pasta intake in healthy male subjects, indicating that the intestinal barrier function was improved [9]. In a case study fecal zonulin has shown to be significantly decreased by eight weeks Ecologic^®^ 825 supplementation in subjects who had an elevated zonulin level (>30 ng/ml) at baseline [27]. Another randomized, double-blind, placebo controlled trial has shown a decrease of fecal zonulin levels by 14 weeks of probiotic mix Ecologic^®^ Performance supplementation in trained men [28]. It should be noted, however, that we did not measure fecal zonulin levels, and thus the outcomes of this study and the present are not directly comparable.

Studies investigating effects of probiotics and prebiotics on immune modulation by determining cytokines in healthy volunteers have reported variable outcomes. A twelve-week intervention with a multispecies probiotic containing *L*. *acidophilus*, *B*. *lactis and B*. *bifidum* combined with FOS, showed a significant decrease in proinfammatory cytokines IL-6 and IL-1β when peripheral blood mononuclear cells were isolated and cultured *ex vivo* in presence of lipopolysaccharide [29]. In a randomized, double-blind, placebo-controlled trial three different probiotic strains were supplemented for 30 days. Serum levels of anti-inflammatory cytokines IL-4 and IL-10 significantly increased in subjects that consumed with *L*. *rhamnosus*. Subjects receiving *L*. *rhamnosus* and *L*. *paracasei* showed a significantly increased IL-10/IL-12 ratio, which is considered as an anti-inflammatory index. Moreover, *L*. *rhamnosus* decreased proinflammatory index TNF-α/IL-10 ratio [30]. *L*. *salivarus* supplementation for four weeks has also been shown to significantly increase plasma IL-10 levels [31]. In contrast, no differences in cytokine expression were found in a double-blind, placebo-controlled study after two months of *L*. *reuteri* supplementation [32]. Also six weeks *Bacteroides xylanisolvents* supplementation did not affect inflammatory markers IL-6, interferon-γ and C-reactive protein levels [33]. Furthermore, four weeks prebiotic β2–1 fructan supplementation did not alter cytokine levels in *in vitro* restimulated blood [34]. Taken together, previous studies on effects of probiotic and prebiotic supplementation on cytokine production *in vivo* did not provide consistent results. No changes in plasma cytokine- and chemokine levels as compared to baseline values have been observed in the present study. In future studies it might be considered to measure cytokine production after *in vitro* stimulation of peripheral blood mononuclear cells [35].

In this study symptom diaries were completed at baseline and during 14 days of supplementation with synbiotic or control. Average symptom scores were low, and ranged between 1.0 and 1.7 on a five-point Likert scale in both groups. No significant differences were observed in average symptom scores between control and Ecologic^®^ 825/FOS P6 intervention. However, Ecologic^®^ 825/FOS P6 supplementation increased stool frequency significantly. Several studies in healthy adults showed that *L*. *salivarius* [31], *L*. *rhamnosus* combined with *L*. *paracasei* [36], *L*. *acidophilus* combined with lactitol [37], *L*. *gasseri* combined with *L*. *coryniformis* [38], FOS [39] and arabinoxylan oligosaccharides [40] significantly increased stool frequency in healthy adults. In contrast, *L*. *casei Shirota* has been shown to decrease stool frequency in healthy adults with soft stools at baseline [41]. In another study, three weeks of *L*. *rhamnosus* intake did not influence stool frequency [42]. A stool frequency considered as normal may vary between one bowel movement per three days to three bowel movements per day. In both intervention groups of our study, stool frequency was within this range. We found evidence that supplementation with the synbiotic Ecologic^®^ 825/FOS P6 increases stool frequency, possibly through acceleration of transit or through other mechanisms. In this respect it will be interesting to evaluate the effects of the synbiotic Ecologic^®^ 825/FOS P6 in subjects with symptomatic constipation. Overall, intake of synbiotic Ecologic^®^ 825/FOS P6 was well tolerated by human subjects in our study.

The generalisability of this study across populations and situations is high, as healthy volunteers were recruited by broad in- and exclusion criteria, and subjects maintained there habitual lifestyle. Moreover, we used a stressor protocol to mimic a compromised state, as for instance in IBD or celiac patients [43]. However, some limitations should be considered. Firstly, although the study was executed as a double-blind randomized controlled study, it had a parallel design. A cross-over study would have allowed comparison within subjects but also is prone to carry-over effects and would have extended the participation period for individual participants considerably, which hampers study compliance and completion rate. Secondly, we assumed that habitual diet in general, and fibre intake more specifically, was the same before and during intervention but we did not control for that. In future research a run-in period of for instance two weeks, in which subjects consume a standardized diet, should be considered in order to correct for differences in dietary fibre intake [9]. Thirdly, the intervention period of two weeks may have been too short to demonstrate significant changes in intestinal permeability. Forthly, we chose to combine Ecologic^®^ 825 with FOS P6 with the assumption to stimulate selective growth and activity of the probiotic strains. This assumption was based on *in vitro* pilot data, not on *in vivo* human data. Theoretically although unlikely, it may be that *in vivo* the mix of pro- and prebiotics may not enhance but possibly counteract beneficial strain specific effects. Fifth, a significant gender dysbalance was present between intervention groups. Finally, baseline urinary sucrose and lactulose excretions were significantly increased after indomethacin challenge in the control group but not in the Ecologic^®^ 825/FOS P6 group. However, the lactulose/rhamnose ratio and sucralose/erythritol ratio are the most accurate parameters of intestinal permeability, because these parameters correct for pre-absorption factors such as gastric emptying, dilution by secretion and intestinal transit time, and post-absorption factors such as systemic distribution and renal clearance affecting both molecules of the ratio equally [44]. Hence, these most important parameters were equally affected by indomethacin in the control and Ecologic^®^ 825/FOS P6 intervention groups.

In conclusion, two weeks Ecologic^®^ 825/FOS P6 supplementation did not reduce intestinal permeability under basal and under indomethacin-induced stressed conditions in healthy adults. Furthermore, two weeks Ecologic^®^ 825/FOS P6 supplementation did not alter immune function and gastrointestinal symptoms in healthy adults. Stool frequency was increased by two weeks Ecologic^®^ 825/FOS P6 supplementation.

## Supporting Information

S1 File Study Protocol (PDF)

S2 File CONSORT Checklist (DOC)

S3 File Manuscript data. (XLSX)
