# Supplementary material for: Effects of Supplementation of the Synbiotic Ecologic® 825/FOS P6 on Intestinal Barrier Function in Healthy Humans: A Randomized Controlled Trial
Source: PLoS One. 2016 Dec 9;11(12):e0167775. doi: 10.1371/journal.pone.0167775 (PMC5147956; doi:10.1371/journal.pone.0167775)
Supplement: S1 File — (PDF) [file pone.0167775.s001.pdf]

RESEARCH PROTOCOL

September 24, 2013

The effects of the synbiotic Ecologic 825/scFOS on intestinal barrier function and immune modulation

**Protocol ID**	
**Short title**	**CRIB**
**EudraCT number**	
**Version**	**2**
**Date**	**September 24, 2013**
**Coordinating investigator/project leader**	Dr. Freddy J Troost<br>T: +31(0)43 3884296<br>E: f.troost@maastrichtuniversity.nl<br>div. Gastroenterology-Hepatology; dept. Internal Medicine<br>Maastricht University Medical Centre<br>Maastricht, The Netherlands
**Principal investigator(s) (in Dutch: hoofdonderzoeker/ uitvoerder)**<br>***<Multicenter research: per site>***	Dr. Freddy J Troost<br>T: +31(0)43 3884296<br>E: f.troost@maastrichtuniversity.nl<br>div. Gastroenterology-Hepatology; dept. Internal Medicine<br>Maastricht University Medical Centre<br>Maastricht, The Netherlands
**Sponsor (in Dutch: verrichter/opdrachtgever)**	***Maastricht University***<br>Prof.dr. A.A.M. Masclee<br>div. Gastroenterology-Hepatology; dept. Internal Medicine<br>Maastricht University Medical Centre<br>Maastricht, The Netherlands
**Subsidising party**	***Food and Nutrition Delta***

	Nieuwe Kanaal 9a<br>*6709 PA Wageningen*<br>*Postbus 450*<br>*6700 AL Wageningen*<br>*T +31 317 487258*<br>*E info@foodnutritiondelta.nl*
**Independent expert (s)**	Dr. Jeroen Kooman, Nefrologie<br>Department of Internal Medicine<br>Maastricht University Medical Centre<br>Maastricht, The Netherlands
**Laboratory sites <*if applicable*>**	***Not applicable***
**Pharmacy <*if applicable*>**	***Not applicable***

PROTOCOL SIGNATURE SHEET

Name	Signature	Date
**Sponsor or legal representative:**<br>**Prof.dr. Ad Masclee**<br>**Head of division Gastroenterology-Hepatology**<br>**Maastricht University Medical Centre**		
**[Coordinating Investigator/Project leader/Principal Investigator]:**<br>***Dr. F.J. Troost***<br>***Assistant professor Gastroenterology-Hepatology***<br>**Maastricht University Medical Centre**		

TABLE OF CONTENTS

1. INTRODUCTION AND RATIONALE....10
1.1 Background....10
2. OBJECTIVES....12
3. STUDY DESIGN....13
4. STUDY POPULATION....15
4.1 Population (base)....15
4.2 Inclusion criteria....15
4.3 Exclusion criteria....15
4.4 Sample size calculation....16
5. TREATMENT OF SUBJECTS....17
5.1 Investigational product/treatment....17
5.2 Use of co-intervention (if applicable)....17
5.3 Escape medication (if applicable)....17
6. INVESTIGATIONAL PRODUCT....18
6.1 Name and description of investigational product(s)....18
6.2 Summary of findings from non-clinical studies....18
6.3 Summary of findings from clinical studies....18
6.4 Summary of known and potential risks and benefits....18
6.5 Description and justification of route of administration and dosage....19
6.6 Dosages, dosage modifications and method of administration....19
6.7 Preparation and labelling of Investigational Medicinal Product....19
6.7.1 Description of the synbiotic (Ecologic® 825/scFOS)....19
6.7.2 Production of the synbiotic....20
6.7.3 Probiotic supplement preparation.... **Fout! Bladwijzer niet gedefinieerd.**
6.7.4 Labelling....22
6.8 Drug accountability....22
7. NON-INVESTIGATIONAL PRODUCT....23
7.1 Name and description of non-investigational product(s)....23
7.2 Summary of findings from non-clinical studies....23
7.3 Summary of findings from clinical studies....23
7.4 Summary of known and potential risks and benefits....23
7.5 Description and justification of route of administration and dosage....23
7.6 Dosages, dosage modifications and method of administration....23
7.7 Preparation and labelling of Non Investigational Medicinal Product....24
7.8 Drug accountability....24
8. METHODS....25
8.1 Study parameters/endpoints....25
8.1.1 Main study parameter/endpoint....25
8.1.2 Secondary study parameters/endpoints (if applicable)....25
8.1.3 Other study parameters (if applicable)....25
8.2 Randomisation, blinding and treatment allocation....25

8.3 Study procedures 26
8.3.1 Catheter positioning 26
8.3.2 Gut permeability test 27
8.3.3 Blood sampling 28
8.3.4 Questionnaire 28
8.3.5 Microbiota analyses 28
8.4 Withdrawal of individual subjects 30
8.5 Replacement of individual subjects after withdrawal 30
8.6 Follow-up of subjects withdrawn from treatment 30
9. SAFETY REPORTING 31
9.1 Section 10 WMO event 31
9.2 AEs, SAEs and SUSARs 31
9.2.1 Adverse events (AEs) 31
9.2.2 Serious adverse events (SAEs) 31
9.3 Follow-up of adverse events 32
10. STATISTICAL ANALYSIS 32
11. ETHICAL CONSIDERATIONS 33
11.1 Regulation statement 33
11.2 Recruitment and consent 33
11.3 Objection by minors or incapacitated subjects (if applicable) 33
11.4 Benefits and risks assessment, group relatedness 33
11.5 Compensation for injury 33
11.6 Incentives (if applicable) 34
12. ADMINISTRATIVE ASPECTS, MONITORING AND PUBLICATION 35
12.1 Handling and storage of data and documents 35
12.2 Amendments 35
12.3 Annual progress report 36
12.4 End of study report 36
12.5 Public disclosure and publication policy 36
13. STRUCTURED RISK ANALYSIS 37
13.1 Potential issues of concern 37
13.2 Synthesis 38
14. REFERENCES 39

LIST OF ABBREVIATIONS AND RELEVANT DEFINITIONS

CCMO	Central Committee on Research Involving Human Subjects
CFU	colony forming units
LAB	Lactic acid bacteria
L. plantarum	*Lactobacillus* plantarum
METC	Medical research ethics committee (MREC); in Dutch: medisch ethische toetsing commissie (METC)
MUMC	Maastricht University Medical Centre
(S)AE	Serious Adverse Event
scFOS	short chain fructo-oligosaccharide
Sponsor	The sponsor is the party that commissions the organization or performance of the research, for example a pharmaceutical company, academic hospital, scientific organization or investigator. A party that provides funding for a study but does not commission it is not regarded as the sponsor, but referred to as a subsiding party.
SUSAR	Suspected Unexpected Serious Adverse Reaction
WMO	Medical Research Involving Human Subjects Act (in Dutch: Wet Medisch wetenschappelijk Onderzoek met mensen)

SUMMARY

Rationale: In the present pilot study, we will study the effects of a novel synbiotic, which is a mix of probiotics (Ecologic 825) in the presence of a prebiotic (scFOS), on mucosal integrity, overall microbiota changes along the GI-tract and the mucosal immune response. We hypothesize that the synbiotic Ecologic 825/scFOS will significantly affect the intestinal permeability and modulate the immune system in humans.

Objective: The primary objective of this study is to assess the effects of the symbiotic *Ecologic 825*/scFOS on intestinal epithelial permeability.

This study has four secondary objectives:

1. The first secondary objective of this study is to study the microbiota composition in healthy humans at three different points along the small intestinal tract (duodenum, jejunum, ileum) using culture–independent approaches.
2. The second secondary objective of this study is to assess the impact of supplementation of the synbiotic Ecologic® 825/scFOS on the population dynamics and functionality of the microbiota along the gastrointestinal tract (duodenum, jejunum, ileum and feces) using culture–independent approaches. This includes potential stimulation of CRIB, as determined by 16S rRNA gene copies counts in ileum fluid.
3. The third secondary objective of this study is to assess the effects of the synbiotic Ecologic® 825/scFOS on immune modulation, by determining the levels of several cytokines and chemokines in blood plasma (TNFa, IL-1b, IL-6, IL-8, IL-17, MCP-1 and MIP-1a).
4. The fourth secondary objective of this study is to determine whether ileal CRIB counts associate with any of the parameters mentioned under the primary objective, and under the third secondary objective.

Study design: The design conforms to a randomized double-blind placebo-controlled parallel design.

Study population: Healthy volunteers, age between 18 and 65 yrs, will participate in this study.

Intervention (if applicable): One group receives synbiotic supplements twice daily, and a second group receives placebo twice daily. Before and after the supplementation period, several measurements will take place.

Main study parameters/endpoints: The main study parameter is the change in urinary sugar excretion ratio before and after the intervention period.

Nature and extent of the burden and risks associated with participation, benefit and group relatedness: A total amount of 8 mL venous blood will be sampled. On two occasions, an intestinal feeding catheter will be placed with the catheter tip positioned in the ileum, to aspirate intestinal contents. This will occur under fluoroscopic control. The radiation exposure is minimal and induces no health risk to the healthy subjects. Urine will have to be collected on 4 different occasions. On two occasions, participants will have to ingest a small dosage of the non-steroidal anti-inflammatory drug indomethacin. Participants will have to visit the university facility on 9 occasions. A participant will spend 12.5 hrs at the university facility over the entire study period. The synbiotics and probiotics, which will be offered to participants are safe for human consumption.

1. INTRODUCTION AND RATIONALE

The present investigation is based on the findings of recently performed human experiments at the MUMC Maastricht (MEC 11-2-011, MEC 05-118 and MEC 03-196), and on the established association between the presence of the *commensal rat ileum bacterium* (CRIB) and infectious complications in rats suffering from acute pancreatitis (1). In the human studies, the effects of oral and intraluminal administration of probiotic bacterial strains on gene expression, protein expression and intestinal permeability in the mucosa of the small intestine were examined.

1.1 Background

In a previous research project, we have identified a novel commensal bacterium present in the ileum of rats. We have provisionally named this novel bacterial species CRIB, which stands for commensal rat ileum bacterium. It is an interesting commensal bacterium since its presence in the ileum of rats has been associated with a decreased severity of acute pancreatitis and associated sepsis (1). More specifically, a higher relative abundance of CRIB in the small intestine (ileum) of the rats was significantly correlated with improved disease pathology, decreased duodenal bacterial overgrowth, and reduced bacterial translocation to remote organs. Severe acute pancreatitis results in gut microbiota dysbiosis and disturbed gut barrier integrity, causing infections and in an end stage of disease sepsis and subsequent death. It has been demonstrated in a rat model that during acute pancreatitis bacterial translocation occurs from the small intestine rather than from the colon. Interestingly, rats which carried this commensal bacterium in their ileum were protected from sepsis and subsequent mortality, which suggest a potential role of this in strengthening the small intestinal barrier. In addition, the relative abundance of CRIB was correlated with altered plasma cytokine levels during acute pancreatitis. The ileum, the site where CRIB is most abundant, is also the main site for interaction of commensal microbiota with the host immune system. This is reflected by the fact that the Peyer's patches, organized lymphoid tissue important for immune surveillance and initiating of immune responses in the gut, are mainly located in the ileum. This is suggests that CRIB might confer protective effects by modulation of the mucosal immune system.

We have demonstrated that CRIB can be a dominant member of the ileal microbiota in rats. However, so far we only have been able to test a limited number of samples derived from the small intestine of humans. Ileostoma effluent samples of 6 individuals have been tested. Four of the six individuals had detectable amounts of CRIB. Based on these observations, we have reason to believe that CRIB can also be a contributing member of the ileal microbiota in

humans. In this project, we will study whether we can modulate the amount of CRIB in the small intestine by 14-days supplementation with the synbiotic Ecologic® 825/short chain fructo-oligosaccharide (scFOS).

The synbiotic (Ecologic® 825/scFOS) used in this study is a combination of a multispecies probiotic product (Ecologic® 825) with a prebiotic (scFOS). The multispecies probiotic powder contains 8 bacterial strains. These strains are selected based on their potential to beneficially affect several parameters involved in intestinal permeability and immune modulation (see section 3.2.5 'The synbiotic (Ecologic® 825/scFOS)' for details on the strain selection). So far, already positive results have been seen of Ecologic® 825 on intestinal barrier function in pouchitis patients (randomized controlled study; reference ethics commitee 109-08; Regionala etikprövningsnämnden i Linköping, Sweden) and in patients with health problems related to intestinal barrier dysfunction. Besides the probiotic component Ecologic® 825, also a prebiotic component is included. Prebiotics are defined as "a non-digestible food ingredient that beneficially affects the host by selectively stimulating the growth and/or activity of one or a limited number of bacteria in the colon that can improve the host health". The synbiotic product used in this study will contain a daily dosage of 10 grams of prebiotic. It is a short-chain fructo-oligosaccharide (scFOS), which is a mixture of oligosaccharides consisting of short chains of glucose linked to fructose units (degree of polymerization 3-5). From all prebiotics available currently, scFOS has been selected as prebiotic of choice since it is able to stimulate both the growth and activity of the probiotic strains in Ecologic® 825 as well as the growth of CRIB *in vitro*. By combining Ecologic® 825 with the prebiotic scFOS we form a synbiotic product that is able to exert a possible prebiotic effect by stimulating the growth of the individual probiotic strains, as well as support the growth of CRIB.

Effects of supplementation of a moderate amount of synbiotics are generally small and are difficult to assess in the healthy gut. The effects due to synbiotic supplementations may be more pronounced in a compromised gut. In the present study, the non-steroidal anti-inflammatory drug indomethacin is used to cause a, highly standardized, reversible damage to the healthy small intestine. Indomethacin is particularly useful because it mildly disturbs the integrity of the intestinal mucosal barrier in all healthy volunteers, as was shown repeatedly in many previous studies. The indomethacin protocol to obtain a reversibly compromised gut, as will be applied in the present investigation, has previously been approved by the METC azM/UM (MEC 00-047, MEC 11-2-011).

In the present pilot study, we will study the effects of a mix of probiotics (Ecologic 825) in the presence of a prebiotic (scFOS) on stimulation of endogenous CRIB, the epithelial integrity, overall microbiota changes along the GI-tract and the mucosal immune response.

2. OBJECTIVES

Primary Objective: The primary objective of this study is to assess the effects of the symbiotic *Ecologic 825*/scFOS on intestinal epithelial permeability.

Secondary Objective(s): This study has four secondary objectives:

1. The first secondary objective of this study is to study the microbiota composition in healthy humans at three different points along the small intestinal tract (duodenum, jejunum, ileum) using culture–independent approaches.
2. The second secondary objective of this study is to assess the impact of supplementation of the synbiotic Ecologic® 825/scFOS on the population dynamics and functionality of the microbiota along the gastrointestinal tract (duodenum, jejunum, ileum and feces) using culture–independent approaches. This includes potential stimulation of CRIB, as determined by 16S rRNA gene copies counts in ileum fluid.
3. The third secondary objective of this study is to assess the effects of the synbiotic Ecologic® 825/scFOS on immune modulation, by determining the levels of several cytokines and chemokines in blood plasma (TNFa, IL-1b, IL-6, IL-8, IL-17, MCP-1 and MIP-1a).
4. The fourth secondary objective of this study is to determine whether ileal CRIB counts associate with any of the parameters mentioned under the primary objective, and under the third secondary objective.

3. STUDY DESIGN

In this project, the effects of a synbiotic will be investigated. The design conforms to a randomized double-blind placebo-controlled parallel design. Each subject will have to undergo one of two different experimental periods. During the test period, a symbiotic (Ecologic 825/ scFOS) or placebo will be supplemented. The intervention per subject will be decided by a randomization procedure.

Volunteers will be recruited using posters at the local libraries and student frequented areas and the website www.digi-prik.nl. Initial information will be provided verbally, the written information brochure will be send by regular mail. If the volunteer is interested, an appointment will be made for a medical screening, at least one week after receipt of the written information. After additional information is given, the volunteer can choose to sign the Informed Consent form (a copy will be provided for the volunteer). After giving written informed consent, the screening will take place. If the volunteer seems likely to qualify as a candidate, a medical screening, involving a standardised examination by the medical researcher/physician will be performed. A pregnancy test will be performed in all female subjects. Physical health will be assessed by means of standard physical examination. These examinations will require a half hour-long visit to the University Hospital. Should there be any abnormalities regarding the participant’s medical-physiological state, the medical researcher/physician executing the screening will advise the participant to consult a specialist and on request will contact the relevant specialist. Subjects will be informed in case the abnormalities (such as a positive pregnancy test) are found during the screening which excludes participation in the study.

After enrollement, during a 17-days period, all patients will consume supplements containing either 1) $7.5 * 10^9$ CFU probiotics and 5 g scFOS/supplement; 2) placebo during breakfast and during dinner. Prior to the start of the supplementation period, several baseline measurements will be done. First, the microbial composition of the intestinal fluid in the proximal- mid- and distal small intestine will be determined. To enable this, a naso-ileal sampling catheter will be placed with the catheter tip located in the ileum. After successful positioning of the catheter, 10 ml of intestinal fluid will be sampled from each location by aspiration using a 20-ml syringe. After sampling, the catheter will be removed by gently retracting the catheter. On the same day, a blood sample will be obtained to measure parameters of the immune system, and a fecal donation will be handed in by the subject, to determine the effects of the intervention on microbial composition of feces. On the next morning, small intestinal permeability will be assessed non-invasively by a sugar recovery test. Two days after this first permeability assessment, intestinal permeability in a stressed condition will be determined using an indomethacin challenge. The indomethacin challenge

will compromise the gut, as is explained in the background section of this protocol. These two subsequent measurements of intestinal permeability serve to obtain baseline permeability values as references for the intervention values. After a 14-days supplementation period, this whole procedure will be performed for a second time conform protocol prior to intervention: positioning of a naso-ileal sampling catheter, sampling of small intestinal fluid on the next day together with collection of blood and fecal samples, followed by a permeability test the next morning and a last permeability test under stressed conditions two days later. Gastrointestinal symptoms and feelings of well-being will be scored every second day prior to sleeping, by completing a symptom diary.

4. STUDY POPULATION

4.1 Population (base)

Healthy volunteers will be recruited to participate in this study.

4.2 Inclusion criteria

In order to be eligible to participate in this study, a subject must meet all of the following criteria:

1) Based on medical history and previous self-admitted examination, no gastrointestinal complaints can be defined.
2) Age between 18 and 65 years
3) BMI between 20 and 30 kg/m^2

4.3 Exclusion criteria

A potential subject who meets any of the following criteria will be excluded from participation in this study:

1) History of severe cardiovascular, respiratory, urogenital, gastrointestinal/ hepatic, hematological/immunologic, HEENT (head, ears, eyes, nose, throat), dermatological/connective tissue, musculoskeletal, metabolic/nutritional, endocrine, neurological diseases, allergy, major surgery and/or laboratory assessments which might limit participation in or completion of the study protocol.
2) Use of medication, including vitamin supplementation, except oral contraceptives, within 14 days prior to testing
3) Administration of probiotic supplements, investigational drugs or participation in any scientific intervention study which may interfere with this study (to be decided by the principle investigator), in the 180 days prior to the study
4) Use of antibiotics in the 90 days prior to the study
5) Abdominal surgery interfering with gastrointestinal function, upon judgment of the principle investigator)
6) Pregnancy, lactation
7) Excessive alcohol consumption (>20 alcoholic consumptions per week)
8) Smoking
9) Blood donation within 3 months before or after the study period
10) Self-admitted HIV-positive state
11) History of any side effects towards intake of pro- or prebiotic supplements of any kind.

4.4 Sample size calculation

Considering the primary outcome of this present study, which is the recovery of the permeability markers (sugars), the following calculations were executed in order to determine sample sizes.

Recent work from a group at MUMC, applying the same method to assess small intestinal permeability in the presence and absence of indomethacin in dosages equal to those proposed in the presednt protocol, and using the same laboratory for the sample analyses (4) showed that the urinary lactulose recovery after indomethacin challenge was 15.2 ± 2.1 µmol.

Sample size was determined using PS Power and Sample Size Calculation version 3.0.43.

$\alpha = 0.05$
$\delta = 3.04$ (20%)
$\sigma = 2.1$
$m = 1$

In order to detect a difference of 20% in the lactulose excretion between the intervention groups, at a power of 80% and a significance level of 5%, 9 completers per group are needed. To anticipate on possible drop-outs, a total of 20 subjects will be included in this study.

TREATMENT OF SUBJECTS

4.5 Investigational product/treatment

The synbiotic Ecologic® 825/short chain fructo-oligosaccharide (scFOS) will be supplied to healthy volunteers. The multispecies probiotic powder Ecologic® 825 contains 8 bacterial strains: *Bifidobacterium bifidum* (W23), *B. lactis* (W51), *B. lactis* (W52), *Lactobacillus acidophilus* (W22), *L. casei* (W56), *L. paracasei* (W20), *L. plantarum* (W62), *L. salivarius* (W24) and *Lactococcus lactis* (W19). Supplements will be supplied in a duo-sachet; one sachdet contains the Ecologic® 825, while the other sachet contains the scFOS.

In a second study arm, volunteers will receive placebo; the duo sachet will contain the carrier-material as included in Ecologic 825 except for the probiotic strains. The other sachet will contain 5 gram of maltodextrin, which has a similar appearance as the scFOS, but not the prebiotic properties.

4.6 Use of co-intervention (if applicable)

Not applicable.

4.7 Escape medication (if applicable)

Not applicable.

5. INVESTIGATIONAL PRODUCT

5.1 Name and description of investigational product(s)

- The synbiotic Ecologic® 825/short chain fructo-oligosaccharide (scFOS);
- Placebo (carrier material/maltodextrin)

5.2 Summary of findings from non-clinical studies

Not applicable; see section 5.3.

5.3 Summary of findings from clinical studies

The present investigation is based on the findings of recently performed human experiments at the MUMC Maastricht (MEC 11-2-011, MEC 05-118 and MEC 03-196), and on the established association between the presence of the *commensal rat ileum bacterium* (CRIB) and infectious complications in rats suffering from acute pancreatitis (1). In the human studies, the effects of oral and intraluminal administration of probiotic bacterial strains on gene expression, protein expression and intestinal permeability in the mucosa of the small intestine were examined. The clinical studies are discussed in section 1.1 of this research protocol.

5.4 Summary of known and potential risks and benefits

There are no risks know to be associated with or expected from the suppletion of synbiotics or probiotics by healthy subjects. During this study, intestinal sampling catheters will be placed with the catheter tip located in the small intestine. Catheters will be positioned under fluoroscopic control. The subjects will perceive mild discomfort during the placement of the catheter. The radiation exposure during the positioning of the catheter is minimal (approximately 2-2.5 sec/scan). Each test day starts with checking the position of the tube. The total radiation exposure time of the procedure is estimated to be 20 seconds, equaling a total radiation exposure of 0.2 mSv per volunteer for the total duration of the study, in which the naso-intestinal catheter will have to be positioned twice (at baseline and after the suppletion period). In patients, there is a very small risk on inducing gastrointestinal perforation during catheter positioning. Positioning of the catheter in healthy volunteers does not cause a known health risk. Statistics on this risk in healthy people are not available. The same procedure of positioning this catheter in healthy volunteers has been approved previously by the METC azM/UM previously (MEC 11-3-034).

5.5 Description and justification of route of administration and dosage

The synbiotics, ~~probiotics~~ and placebo will be taken as oral supplements.

5.6 Dosages, dosage modifications and method of administration

The synbiotic supplement contains 7.5 * 10^9 CFU probiotics and 5 g scFOS/supplement (see 6.7 *Synbiotic supplement preparation*);
The placebo supplements contain 3 g of the probiotic carrier material, and 5 g maltodextrin

5.7 Preparation and labelling of Investigational Medicinal Product

5.7.1 Description of the synbiotic (Ecologic® 825/scFOS)

The synbiotic (Ecologic® 825/scFOS) will be kindly supplied by Winclove Probiotics, Amsterdam, the Netherlands. It will be supplied as a duo-sachet containing the probiotic Ecologic® 825 and the prebiotic scFOS separately.

The multispecies probiotic powder contains 8 bacterial strains: *Bifidobacterium bifidum* (W23), *B. lactis* (W51), *B. lactis* (W52), *Lactobacillus acidophilus* (W22), *L. casei* (W56), *L. paracasei* (W20), *L. plantarum* (W62), *L. salivarius* (W24) and *Lactococcus lactis* (W19). The total cell count is 2.5 x 10^9 CFU/gram. These strains are selected based on their potential to beneficially affect several parameters involved in intestinal permeability and immune modulation (based on *in vitro* experiments):

- *B. bifidum* (W23): improvement of barrier function, immune modulation, mast cell inhibition
- *B. lactis* (W51): immune modulation, induction of Treg cells
- *B. lactis* (W52): improvement of barrier function, mast cell inhibition, immune modulation, induction of Treg cells
- *L.acidophilus* (W22): immune modulation
- *L. casei* (W56): improvement of barrier function, mast cell inhibition, immune modulation
- *L. paracasei* (W20): mast cell inhibition, pathogen inhibition
- *L. plantarum* (W62): induction of Treg cells, pathogen inhibition
- *L. salivarius* (W24): improvement of barrier function, mast cell inhibition
- *Lc. lactis* (W19): immune modulation

Besides the 8 bacterial species, Ecologic® 825 consists further of a carrier containing maize starch, maltodextrins, a mineral mix, inulin and FOS. For this study the dose of the scFOS will be increased to prebiotic quantities (10 gram per day). The prebiotic used in this study is a short-chain fructo-oligosaccharide (scFOS).

Twice a day, before breakfast and before going to sleep on an empty stomach, a duo sachet containing either 3 gram of the probiotic and 5 gram of the prebiotic or placebo has to be consumed. The dosage of Ecologic® 825 (7.5 * 10^9 CFU/supplement, equaling 1.5 * 10^{10} CFU/day) is in accordance with most probiotic dosages used in studies and many commercially available products, ranging from 10^9-10^{11} CFU/dose. It is not possible to state a general dose for probiotics; some have shown to be efficacious at lower levels, while other require substantially more. The dosage of Ecologic® 825 is based on prior human studies with both Ecologic® 825 (not published yet) as well as studies with similar probiotic products (5, 6), showing a health benefit and no adverse reactions. The dosage of the prebiotics is in accordance with most probiotic dosages used in studies and many commercially available products, ranging from 2.5 – 20 gram a day. The placebo product will also consist of a duosachet. One sachet will contain 3 gram of the same carrier-material as included in Ecologic 825 except for the probiotic strains. The other sachet will contain 5 gram of maltodextrine, which has a similar appereance as the scFOS, but not the prebiotic properties.

The duo sachets have to be dissolved in a glass containing 100 mL lukewarm water, left for 10 min, stirred, and ingested. The duo sachets will be blinded and coded. The randomization will be performed centrally by Winclove Probiotics. Code numbers can be opened after the end of each study by the coordinating and principal investigator (Dr. F.J. Troost). If a medical problem occurs, for which this information is needed, a contact person at Winclove Probiotics can at all-time be contacted to break the code.

5.7.2 Production of the synbiotic

Winclove Probiotics has been producing multispecies probiotics for over twenty years. The lactic acid bacteria used in this study (*Bifidobacterium bifidum* (W23), *B. lactis* (W51), *B. lactis* (W52), *Lactobacillus acidophilus* (W22), *L. casei* (W56), *L. paracasei* (W20), *L. plantarum* (W62), *L. salivarius* (W24) and *Lactococcus lactis* (W19)) are all commercially available in Ecologic® 825 and also in combination with other strains in other products and all carry the European Union qualified presumption of safety (QPS). The strains present in Ecologic® 825 are normal constituents of the intestinal bacterial community. The product Ecologic® 825 has been applied before in a randomized controlled human study with pouchitis patients (reference 109-08; Regionala etikprövningsnämnden i Linköping, Sweden). and in an open-label study with patients with a disturbed intestinal barrier function. No adverse events or symptoms of any kind were observed by any of the volunteers during these studies. Therefore, no side effects of any kind are expected from the consumption of

this product used in this study. For this study, Ecologic® 825 will be combined a prebiotic (scFOS) in a duo sachet containing 3 gram of the probiotic and 5 gram of the prebiotic (or placebo). This specific prebiotic has been used in previous human studies, showing a health benefit and no adverse reactions.

In the Netherlands probiotics are considered to be food or food supplements and therefore have to be produced under HACCP regulations, which is the Dutch regulation system for safety and hygiene in food and food supplement. All components of Ecologic® 825 are legally admitted as food additives or food components. Both probiotics and placebos, produced for studies, are send for independent analysis of contamination to the Institut für Mikroökologie in Herborn, Germany.

In the report of a European workshop on the safety aspects of lactic acid bacteria used as probiotics, Adams concluded that the available evidence does not indicate any health risk posed by ingestion of lactic acid bacteria (7). The risk of infection is very low and in nearly all cases the source of the infecting organism appeared to be the human commensal microbiota rather than food products. In the PROPATRIA trial where the effect of probiotic prophylaxis in patients with severe acute pancreatitis was assessed (8), intake was associated with an increased risk of mortality due to bowel ischemia. Though the exact mechanism cause is unknown, it was thought to be a combination of enteral nutrition with supplements potentially increasing oxygen demand in critically ill patients, who already were having multi-organ failure (8, 9). In the present study the synbiotic product will be given orally to healthy volunteers, who are not critically ill nor will be fed enterally. Combined with the long history of safe consumption of such products in the general population, of which the study group is a representation and the fact that these probiotic strains have been given orally in several other clinical studies without adverse effects, no serious side effects or adverse events are expected.

All products will be prepared in the food-grade facility of Winclove Probiotics:

Winclove Probiotics B.V.
Hulstweg 11
1032 LB Amsterdam
The Netherlands
020 4350235

The HACCP statement of the production facility, a product description, a flowchart of the production process, a 'sample taking and analysis' procedure, a 'storage and packaging' statement, an example of a certificate of analysis (the actual certificate of analysis can only be provided after production of the actual batch of test products), and an example

of a laboratory assignment to start production of the test products are provided in the appendices.

5.7.3 Labelling

The package will contain the following information:

- Study code (CRIB)
- Subject code (‘PP 1’, ‘PP 2’ and number of entrance in the study), thereby assigning the group blindly. The code is linked with the name, address, date of birth and telephone number of the subject.
- Date and time of intake
- Expiration date

5.8 Drug accountability

The test products will be shipped by delivery courier to the Maastricht University Medical Centre. Products will be labelled according to the randomization scheme, and stored at the Metabolic Research Unit Maastricht.

6. NON-INVESTIGATIONAL PRODUCT

6.1 Name and description of non-investigational product(s)

The non-steroidal anti-inflammatory drug will be orally administered conform standard protocol to induce a reversible increase in intestinal permeability. Indomethacin is particularly useful for this purpose because it mildly disturbs the integrity of the intestinal mucosal barrier in all healthy volunteers, as was shown repeatedly in many previous studies. The indomethacin protocol to obtain a reversibly compromised gut, as will be applied in the present investigation, has previously been approved by the METC azM/UM (MEC 00-047, MEC 11-2-011). Indomethacin is widely in use due to its anti-inflammatory capacity. Product details are provided in the annex.

For the gut permeability test, subjects will ingest the sugar probes sucrose, lactulose, rhamnose, sucralose and erythritol. All sugars are food grade and suitable for human consumption. Details are provided in the annexes.

6.2 Summary of findings from non-clinical studies

Not relevant. Volunteers will not be treated with the non-investigational products.. The indomethacin and the sugar probes will be used in a single dosage, as part of the scientific protocol. Treatment with indomethacin for clinical purposes takes place over a prolonged period of time.
The inert sugar probes will be supplied in a single dosage, in order to determine the urinary excretion.
All products are safe for human use.

6.3 Summary of findings from clinical studies

See 6.2.

6.4 Summary of known and potential risks and benefits

See 6.2.

6.5 Description and justification of route of administration and dosage

These compounds are meant to be taken orally.

6.6 Dosages, dosage modifications and method of administration

All dosages are well within the acceptable daily intake level.

6.7 Preparation and labelling of Non Investigational Medicinal Product

Indomethacin is a common over-the-counter drug, and will be supplied by apotheek van Thoor, Maastricht, The Netherlands. It will be labelled as follows;

The label will contain the following information:

- Study code (CRIB)
- Subject code ('PP 1', 'PP 2' and number of entrance in the study), thereby assigning the group blindly. The code is linked with the name, address, date of birth and telephone number of the subject.
- Date and time of intake
- Expiration date

The sugar probes will be prepared at the Metabolic Research Unit Maastricht. After preparation, the package will be labelled.
The label will contain the following information:

- Study code (CRIB)
- Subject code ('PP 1', 'PP 2' and number of entrance in the study), thereby assigning the group blindly. The code is linked with the name, address, date of birth and telephone number of the subject.
- Date and time of intake
- Expiration date

6.8 Drug accountability

Subjects will receive the non-investigational products directly from the investigator. They will all be consumed during the course of the study. In case of subject drop-out, the products will be collected by the investigator.

7. METHODS

7.1 Study parameters/endpoints

7.1.1 Main study parameter/endpoint

Sugar recovery in urine, as indicator of intestinal permeability (see section 3.3.3 *Gut permeability test*)

7.1.2 Secondary study parameters/endpoints (if applicable)

- Measurements in blood at baseline and after the supplementation period

- Biomarkers of immune modulation; plasma levels of TNFa, IL-1b, IL-6, IL-8, IL-17, MCP-1 and MIP-1a (see section 3.3.4 '*Blood sampling*')

- Measurements in small intestinal fluid (duodenum/jejunum/ileum) and feces

- Relative abundance of CRIB (see section 3.3.5 '*Microbiota analyses')*
- Microbial diversity and population dynamics (see section 3.3.5 '*Microbiota analyses)*
- *In situ* gene expression of the microbiota (see section 3.3.5 '*Microbiota analyses')*

- Questionnaire at baseline, during and after the supplementation period

- The symptom diary (see section 3.3.6 '*Questionnaire*')

7.1.3 Other study parameters (if applicable)

Not applicable

7.2 Randomisation, blinding and treatment allocation

Subjects will be randomized in a double-blind parallel fashion to one of the following experimental conditions:

1. Synbiotic (Ecologic 825/scFOS)
2. Placebo

The randomization list will be generated by a co-worker in the same department as the principal investigator, using a publically available procedure through the internet (http://randomizer.org). The investigator has no access to the randomization list that conceals the treatment code. The investigator and the head of the division of Gastroenterology-Hepatology, prof.dr. Ad Masclee, will receive sealed code envelopes, which reveal the treatment in case of medical emergency. The *condition numbers* as allocated by the co-worker who will carry out the randomization are registered by the investigator in the screening and enrolment log. The 'randomization key' will be provided to the investigator after finishing all experimental and statistical procedures.

7.3 Study procedures

< Please give a description of the procedures, techniques, methods and/or tests to be used to assess the defined study parameters/endpoints. Include information on sample volumes. All procedures that subjects undergo must be listed. A schedule of assessments can be helpful >

Figure 1. Schematic representation of the supplementation period.

	baseline				supplementation Ecologic 825/scFOS, LGG or placebo																	
day	-4	-3	-2	-1	0	1	2	3	4	5	6	7	8	9	10	11	12	13	14	15	16	17
	C																	C				
		LS																	LS			
		B																	B			
			S		S	S	S	S	S	S	S	S	S	S	S	S	S	S	S	S	S	
		F																	F			
			P		P															P		P
					IN																	IN

C - Naso-ileal catheter insertion
LS - Luminal fluid samples from duodenum, jejunum and ileum
B - Blood
S - Symptoms diary
F - Feces
P - Permeability (multi-sugar test)
IN - Indomethacin stress test

7.3.1 Catheter positioning

Subjects arrive at the Department of Gastroenterology at 08:00 AM, after an overnight fast of at least 8 hrs. After local anesthesia of the nasal mucosa (using xylocaine 10% spray (Astrazeneca, Zoetermeer, The Netherlands), a 270 cm long silicon 4-lumen 3.0 mm (outer diameter) catheter, with side-holes at 5, 65, and 125 cm proximal to the distal catheter tip, and an inflatable balloon near the distal tip (MUI Scientific, Ontario, Canada, see appendix 'catheter construction'), will be introduced transnasally by a gastroenterologist. Under intermittent fluoroscopic control, the catheter will be placed with the tube tip located in the proximal small intestine directly distal to the stomach, according to the manufacturer's instructions. The catheter is developed and certified with CE certification, for use in humans.
When the distal tip has passed the ligament of Treitz, the balloon is inflated with 10ml of air to enhance catheter progression by peristalsis to the distal ileum. Volunteers will be asked to drink and eat some biscuits to stimulate bowel motility and walk around to have optimal benefit (due to the upright posture) of gravity. In our experience, the duration of the positioning procedure varies from two to six hours, although in rare cases, the catheter may not in place within that time frame. Within 3-5 hours the tip of the catheter usually is 100-120 cm distal of the ligament of Treitz. Position will be checked by fluoroscopy at approximately 15.00 – 16.00 PM, depending on the capacity at the dept of Radiology. If the catheter has successfully positioned (catheter tip in the mid- to distal region of the ileum, according to the gastroenterologist), the balloon will be deflated. If the catheter is not positioned with the tip

located in the target position at around 16.00 PM, a meal will be offered at the department. Volunteers will be instructed to allow the tube to advance until a clearly marked point (190 cm from nostril). From 22.00 hrs onward, only water/tea without sugar or milk are allowed. The volunteers return to the department at 8:00 hours the next day. Next, the position of the tube will be checked using fluoroscopy. After confirmation of successful positioning of the catheter, a 10-mL sample of ileal, jejunal and duodenal fluid will be obtained by aspiration, using a 20-mL syringe. The same procedure of positioning this catheter in healthy volunteers has been approved previously by the METC azM/UM previously (MEC 11-3-034). After successfully obtaining the luminal fluid samples, the catheter will be removed by gently pulling the catheter.

7.3.2 Gut permeability test

Permeability of the small and large intestine in humans will be assessed non-invasively by measuring the urinary excretion ratios of ingested water-soluble, non-degradable tests probes. This barrier function test is based on a comparison of intestinal permeation of a larger molecule (e.g. lactulose) with that of a smaller molecule (e.g. rhamnose). Permeability of the proximal small intestine can be assessed non-invasively by measuring plasma and urinary excretion of sucrose. Furthermore, permeability of the large intestine will be assessed by measuring plasma or urinary excretion of sucralose and erythritol, test probes that are not only non-degradable but also non-fermentable by microbes. For the gut permeability test the subjects will have to ingest a sugar drink (in 100mL tap water) containing 1g sucrose (Van Gilse automatensuiker, Albert Heijn), 1g lactulose (Centrafarm Services, Etten-Leur), 0.5g L-rhamnose (Danisco Sweeteners, Thomson, Illinois, USA), 1g sucralose (Tate and Lyle Sucralose Inc. Reading Great Britain) and 1g erythritol (Cargill Nederland BV) after an overnight fast. All sugar probes used in this test are accepted and validated parameters of integrity of the intestinal barrier, and proved an accurate estimation of mucosal damage. Preparations of the solution will be performed in a food grade kitchen at the Metabolic Research Unit Maastricht.

Urine samples will be obtained to measure sugar recovery. Urine aliquots will be collected in subfractions (0-5 hrs and 5-24 hrs after intake of the sugar markers, respectively) and stored at -80°C until analysis. Sucrose, lactulose, L-rhamnose, sucralose and erithrytol will be determined by fluorescent detection high-pressure liquid chromatography (HPLC). The method was approved previously by the METC azM/UM (MEC 08-2-070, MEC 11-2-011). Additionally, intestinal fatty acid binding protein (iFABP) will be determined by ELISA as described elsewhere (11) as marker of tight junction complex dynamics.

Intestinal permeability will be assessed at baseline, at baseline after an indomethacin challenge and after the 14-days supplementation period in the absence and in the presence of the indomethacin challenge. The indomethacin will be administered in two different dosages. On the evening prior to the permeability test, exactly 9 hrs prior to intake of the multisugar drink, subjects will have to ingest 75 mg of indomethacin. The next morning, exactly 1 hr prior to intake of the multisugar drink, subjects will have to ingest 50 mg indomethacin. After intake of the first indomethacin dosage, no food or beverage intake is allowed, with the exception of water *ad libitum*. The indomethacin protocol is identical to the protocol that has previously been applied and published in studies investigating intestinal permeability in healthy volunteers (12, 13). As those studies showed effects of indomethacin on all individuals tested, whereas no side effects occurred, the same protocol will be applied in this study.

7.3.3 Blood sampling

Blood samples will be collected from an antecubital vein in the fore-arm. Blood will be collected in a single 4-mL BD Vacutainer® K_2EDTA Tube (BD, Breda, The Netherlands) . The tubes will be inverted five times, and centrifuged for 10 minutes at 1000-1300 RCF (g) in a swing bucket centrifuge. After centrifugation, plasma will be stored at -80 °C until determination of the cytokines TNFa, IL-1b, IL-6, IL-8, IL-17, MCP-1 and MIP-1a by MSD-multiplex. This multiplex cytokine assay is a validated method developed for the fast, reliable, reproducible and simultaneous detection of multiple cytokines in the blood plasma of both patients and healthy volunteers, based on antibody detection (14). Changes in cytokine levels will be correlated to changes in microbiota composition and barrier function.

The study protocol includes two blood sampling events; at baseline and after the supplementation period. Thus, a total amount of 8 mL blood will be sampled per volunteer.

7.3.4 Questionnaire

To assess a general comprehension of gastrointestinal symptoms, subjects will have to complete the symptom diary every day, prior to sleeping.

7.3.5 Microbiota analyses

Fecal- and intestinal lumen samples will be subjected to microbiota analysis. Feces will be collected in dedicated feces collection buckets, which will be handed in by the volunteers to the investigator. Intestinal lumen samples will be obtained by aspiration, using the intestinal

Microbiota analyses will be done on small intestinal fluid samples and fecal samples by the Laboratory of Microbiology, Wageningen University.

First, total DNA will be extracted from the small intestinal fluid and fecal samples using the repeated bead-beating method. This method has been optimized and standardized for the extraction of total bacterial DNA from human fecal samples (15).

In order to determine the relative abundance of CRIB, quantitative PCR (qPCR) will be performed on the total DNA extracted from the small intestinal fluid and faecal samples. For this the universal primer set Bact1369/Prok1492 will be used for quantification of total bacterial 16S rRNA gene copies. In addition, for quantification of the CRIB 16S rRNA gene copies the optimized CRIB-specific primer set CRIB-61F/CRIB-192R2 will be used. The relative abundance of CRIB can be calculated by dividing CRIB 16S rRNA gene copies amplified using the primer set CRIB-61F/CRIB-192R2 by total 16S rRNA gene copies amplified using the primer set Bact1369/Prok1492 per µl of isolated DNA.

Total microbiota composition in the small intestinal fluid samples will be determined using barcoded pyrosequencing of 16S rRNA genes as described previously (16). In short, the total DNA extracted from the the small intestinal fluid samples will be amplied using primers (containing sample-specific barcodes targeted) which targetpartof the 16S rRNA genes containing the hypervariable regions V1 and V2. All amplicons will be mixed in equimolar proportions and sequenced by 454 pyrosequencing using Titanium chemistry. The sequences will be binned by samples and subsequently used to determine the microbiota composition. Using the Quantitative Insights Into Microbial Ecology (QIIME) pipeline the diversity of the microbiota and impact of 14-days supplementation of the synbiotic (Ecologic® 825/scFOS) on the microbiota composition and diversity will be determined.

A subset of the small intestinal fluid samples will be used for functional analysis of the microbiota. A metatranscriptomics approach will be performed using strategies that are currently being developed. The metatranscriptomic analyses will provide information about *in situ* gene expression of the small intestinal microbiota and how this is impacted by the 14-days supplementation of the synbiotic (Ecologic® 825/scFOS).

7.4 Withdrawal of individual subjects

Subjects can leave the study at any time for any reason if they wish to do so without any consequences. The investigator can decide to withdraw a subject from the study for urgent medical reasons.

7.5 Replacement of individual subjects after withdrawal

Subjects will not be replaced after withdrawel. In the design of the study, we anticipate a drop-out of maximally 3 volunteers. Hence, we will recruit 3 additional volunteers on top of the required 24 volunteers.

7.6 Follow-up of subjects withdrawn from treatment

Volunteers who dropped out of the study will be followed up by the investigator and by the person who is medically responsible for the study, depending on the reason of the drop-out.

8. SAFETY REPORTING

8.1 Section 10 WMO event

In accordance to section 10, subsection 1, of the WMO, the investigator will inform the subjects and the reviewing accredited METC if anything occurs, on the basis of which it appears that the disadvantages of participation may be significantly greater than was foreseen in the research proposal. The study will be suspended pending further review by the accredited METC, except insofar as suspension would jeopardise the subjects' health. The investigator will take care that all subjects are kept informed.

8.2 AEs, SAEs and SUSARs

8.2.1 Adverse events (AEs)

Adverse events are defined as any undesirable experience occurring to a subject during the study, whether or not considered related to the investigational product / the experimental intervention. All adverse events reported spontaneously by the subject or observed by the investigator or his staff will be recorded.

8.2.2 Serious adverse events (SAEs)

A serious adverse event is any untoward medical occurrence or effect that at any dose:

- results in death;
- is life threatening (at the time of the event);
- requires hospitalisation or prolongation of existing inpatients' hospitalisation;
- results in persistent or significant disability or incapacity;
- is a congenital anomaly or birth defect;
- Any other important medical event that may not result in death, be life threatening, or require hospitalization, may be considered a serious adverse experience when, based upon appropriate medical judgement, the event may jeopardize the subject or may require an intervention to prevent one of the outcomes listed above.

The sponsor will report the SAEs through the web portal *ToetsingOnline* to the accredited METC that approved the protocol, within 15 days after the sponsor has first knowledge of the serious adverse reactions.

SAEs that result in death or are life threatening should be reported expedited. The expedited reporting will occur not later than 7 days after the responsible investigator

has first knowledge of the adverse reaction. This is for a preliminary report with another 8 days for completion of the report.

8.3 Follow-up of adverse events

All AEs will be followed until they have abated, or until a stable situation has been reached. Depending on the event, follow up may require additional tests or medical procedures as indicated, and/or referral to the general physician or a medical specialist. SAEs need to be reported till end of study within the Netherlands, as defined in the protocol

9. STATISTICAL ANALYSIS

Differences between the different interventions will be assessed. Dichotomous variables will be analyzed using the Chi-square test.

Normality of ‘continuous’ variables will be determined by Kolmogorov-Smirnov test. The dependent variables will be analyzed with the Wilcoxon signed-ranked test, when the distribution of the variables is non-parametric. If the variables are normally distributed an analysis of variance will be performed.

The main study parameter, to assess the effects of the symbiotic *Ecologic 825*/scFOS on intestinal permeability, will be statistically analyzed by comparing the effects of the interventions on the changes in small intestinal permeability.

All data obtained in the microbiological part of the study will be imported in the Canoco program which will allow multivariate analysis of ecological data (17). This will result in obtaining links between microbial groups, specific functional pathways and the impact of different environmental factors (individual, supplementation) on the microbiota composition. In addition, the corresponding microbiota gene expression described by the mRNA reads and their originating microbe will be determined. Comparative analyses of gene-expression will be determined by normalizing expression values between samples and quantitative pathway construction based on Ipath platforms and in house scripts.

10. ETHICAL CONSIDERATIONS

10.1 Regulation statement

This study will be conducted according to the principles of the Declaration of Helsinki (*59th WMA General Assembly, Seoul, Korea, October 2008*) and in accordance with the Medical Research Involving Human Subjects Act (WMO).

10.2 Recruitment and consent

A time period of one week is provided to decide whether the volunteer would like to participate. All participants will have to sign an informed consent prior to screening (see appendix toestemmingsverklaring). Participants can, at any time, make an appeal to the independent doctor that has been appointed to this study. Participants will be informed that their decision to participate is totally voluntary and they can withdraw at any time without giving a reason. Participants will have the opportunity to be informed about their individual results and the group results at the end of the study. Consent of participants will be asked for eventual approach in the future for the collection of symptom questionnaires.

10.3 Objection by minors or incapacitated subjects (if applicable)

Not applicable

10.4 Benefits and risks assessment, group relatedness

There will be no direct benefits to the subjects, as a result of participating in this trial. The risk of participation is very low. The subjects will have to invest time, for which they will be compensated;see paragraph 106 ‘Incentives’.
The subjects will perceive mild discomfort during the placement of the catheter. The radiation exposure during the positioning of the feeding tube is minimal (2.5 sec/scan). Each test day starts with checking the position of the tube. The total radiation exposure time of the procedure is estimated to be 20 seconds, equaling a total radiation exposure of 0.2 mSv per volunteer for the total duration of the study, in which the naso-ileal catheter will have to be positioned twice.
Positioning of an intestinal feeding catheter is associated with a risk to cause intestinal perforation. No literature is available on the actual risk of causing perforation in healthy people. This risk is generally considered as nil.

10.5 Compensation for injury

The sponsor/investigator has a liability insurance which is in accordance with article 7, subsection 6 of the WMO.

The sponsor (also) has an insurance which is in accordance with the legal requirements in the Netherlands (Article 7 WMO and the Measure regarding Compulsory Insurance for Clinical Research in Humans of 23th June 2003). This insurance provides cover for damage to research subjects through injury or death caused by the study.

1. € 450.000,-- (i.e. four hundred and fifty thousand Euro) for death or injury for each subject who participates in the Research;
2. € 3.500.000,-- (i.e. three million five hundred thousand Euro) for death or injury for all subjects who participate in the Research;
3. € 5.000.000,-- (i.e. five million Euro) for the total damage incurred by the organisation for all damage disclosed by scientific research for the Sponsor as 'verrichter' in the meaning of said Act in each year of insurance coverage.

The insurance applies to the damage that becomes apparent during the study or within 4 years after the end of the study.

10.6 Incentives (if applicable)

In compensation, participants will receive 600 EUR participating in case of fully completing the study. Proportional amounts will be given in case of incomplete participation.

11. ADMINISTRATIVE ASPECTS, MONITORING AND PUBLICATION

11.1 Handling and storage of data and documents

Data are handled confidentially and anonymously. The privacy of the participants will be guaranteed. All samples and data will be coded in such a way that no personal information about the participants will be available. Each subject will be allocated an individual code which will be visible on biological samples. The code also contains the test number in chronological order and an indicator of baseline ("b") or after the intervention period ("a"). The label of each sample will contain the date of collection. The key of the code is kept by the principal investigator in a locked cabinet, to which only the principal investigator has access. All primary documents and data shall be kept for 15 years after the end of the experimental phase of the study for possible inspection. Samples taken from the participants during the study will be kept for 4 years after the end of the experimental phase of the study for additional analysis. In the informed consent, subjects indicate whether they give consent for storing and keeping these samples, which may be used for additional analyses in the line of the current investigation. Such additional analyses may be determination of new biomarkers, which will be described in internation peer-reviewed papers, in the near future. If subjects deny this consent, all samples will be destructed after carrying out the analyses as described in this protocol. Any additional analysis on samples obtained during the current study will only take place after obtaining approval for an amended protocol by the ethical committee (METC azM/UM).

11.2 Amendments

A 'substantial amendment' is defined as an amendment to the terms of the METC application, or to the protocol or any other supporting documentation, that is likely to affect to a significant degree:

- the safety or physical or mental integrity of the subjects of the trial;
- the scientific value of the trial;
- the conduct or management of the trial; or
- the quality or safety of any intervention used in the trial.

All substantial amendments will be notified to the METC and to the competent authority.

Non-substantial amendments will not be notified to the accredited METC and the competent authority, but will be recorded and filed by the sponsor.

11.3 Annual progress report

The sponsor/investigator will submit a summary of the progress of the trial to the accredited METC once a year. Information will be provided on the date of inclusion of the first subject, numbers of subjects included and numbers of subjects that have completed the trial, serious adverse events/ serious adverse reactions, other problems, and amendments.

11.4 End of study report

The sponsor will notify the accredited METC and the competent authority of the end of the study within a period of 90 days. The end of the study is defined as the last patient's last visit.

In case the study is ended prematurely, the sponsor will notify the accredited METC and the competent authority within 15 days, including the reasons for the premature termination.

Within one year after the end of the study, the investigator/sponsor will submit a final study report with the results of the study, including any publications/abstracts of the study, to the accredited METC and the Competent Authority.

11.5 Public disclosure and publication policy

All trial results, both positive and negative, will be disclosed in agreement with the CCMO statement on publication policy.

Based on the results of this study, at least one publication will be submitted for publication to a peer-reviewed scientific journal. The authorship of the article shall be determined in appropriate consultations based on a considerable contribution to the set-up and execution of the study and an active participation in publication.

This study will be registered in a public trial registry before the first volunteer is recruited.

12. STRUCTURED RISK ANALYSIS

12.1 Potential issues of concern

The subjects will perceive mild discomfort during the placement of the catheter. The radiation exposure during the positioning of the feeding tube is very low (0.2 mSv per volunteer for the total duration of the study).

Positioning of an intestinal feeding catheter is associated with a risk to cause intestinal perforation. No literature is available on the actual risk of causing perforation in healthy people. This risk is generally considered as nil.

a. Level of knowledge about mechanism of action

The synbiotic (Ecologic® 825/scFOS) used in this study is a combination of a multispecies probiotic product (Ecologic® 825) with a prebiotic (scFOS). The multispecies probiotic powder contains 8 bacterial strains. These strains are selected based on their potential to beneficially affect several parameters involved in intestinal permeability and immune modulation (see section 3.2.5 'The synbiotic (Ecologic® 825/scFOS)' for details on the strain selection). So far, already positive results have been seen of Ecologic® 825 on intestinal barrier function in pouchitis patients (randomized controlled study; reference ethics commitee 109-08; Regionala etikprövningsnämnden i Linköping, Sweden) and in patients with health problems related to intestinal barrier dysfunction. Besides the probiotic component Ecologic® 825, also a prebiotic component is included. Prebiotics are defined as "a non-digestible food ingredient that beneficially affects the host by selectively stimulating the growth and/or activity of one or a limited number of bacteria in the colon that can improve the host health". The synbiotic product used in this study will contain a daily dosage of 10 grams of prebiotic. It is a short-chain fructo-oligosaccharide (scFOS), which is a mixture of oligosaccharides consisting of short chains of glucose linked to fructose units (degree of polymerization 3-5). From all prebiotics available currently, scFOS has been selected as prebiotic of choice since it is able to stimulate both the growth and activity of the probiotic strains in Ecologic® 825 as well as the growth of CRIB *in vitro*. By combining Ecologic® 825 with the prebiotic scFOS we form a synbiotic product that is able to exert a possible prebiotic effect by stimulating the growth of the individual probiotic strains, as well as support the growth of CRIB.

b. Previous exposure of human beings with the test product(s) and/or products with a similar biological mechanism

The use of probiotics by consumers is widespread. The specific probiotics used in this study have been consumed by humans previously; see paragraph 13.1.a. The different

probiotic strains have also been consumed by humans. No side effects were reported. Their use is considered to be safe in the amounts used in the current study.

c. Can the primary or secondary mechanism be induced in animals and/or in *ex-vivo* human cell material?
Not applicable; in this study, effects of supplementation of synbiotics and probiotics on humans *in vivo* are assessed.

d. Selectivity of the mechanism to target tissue in animals and/or human beings
Not relevant.

e. Analysis of potential effect
The dosages of synbiotics used in this study are commonly used as food additives. They are considered to be safe for human use.

f. Pharmacokinetic considerations
Not relevant.

g. Study population

Healthy volunteers are recruited.

h. Interaction with other products
The test products are food products. They are normally consumed in combination with a normal human diet. No interaction with other products, posing a health risk to the individual of any kind, are expected.

i. Predictability of effect
The markers chosen in this study have been used previously in scientific studies to the effects of synbiotics. They are suitable to answer the research questions.

j. Can effects be managed?
Not applicable.

12.2 Synthesis

There is a theoretical risk to induce intestinal perforation during the positioning of the catheter. This risk is very low. During catheter positioning, subjects are exposed to radiation. This exposure is also very low. There are no risks associated with the test products in this study. The products are nutritional constituents, and impose no risk to a healthy individual.

13. REFERENCES

1. Gerritsen J, Timmerman HM, Fuentes S, et al. Correlation between protection against sepsis by probiotic therapy and stimulation of a novel bacterial phylotype. Appl Environ Microbiol 2011;77(21):7749-56. doi: 10.1128/AEM.05428-11.
2. de Vos WM. Systems solutions by lactic acid bacteria: from paradigms to practice. Microb Cell Fact 2011;10 Suppl 1:S2. doi: 10.1186/1475-2859-10-S1-S2.
3. Saxelin M, Tynkkynen S, Mattila-Sandholm T, de Vos WM. Probiotic and other functional microbes: from markets to mechanisms. Curr Opin Biotechnol 2005;16(2):204-11. doi: 10.1016/j.copbio.2005.02.003.
4. Van Wijck K, Verlinden TJ, van Eijk HM, Dekker J, Buurman WA, Dejong CH, Lenaerts K. Novel multi-sugar assay for site-specific gastrointestinal permeability analysis: a randomized controlled crossover trial. Clin Nutr. 2013 Apr;32(2):245-51.
5. Bekkali NL, Bongers ME, Van den Berg MM, Liem O, Benninga MA. The role of a probiotics mixture in the treatment of childhood constipation: a pilot study. Nutr J 2007;6:17. doi: 10.1186/1475-2891-6-17.
6. Koning CJ, Jonkers DM, Stobberingh EE, Mulder L, Rombouts FM, Stockbrugger RW. The effect of a multispecies probiotic on the intestinal microbiota and bowel movements in healthy volunteers taking the antibiotic amoxycillin. Am J Gastroenterol 2008;103(1):178-89. doi: 10.1111/j.1572-0241.2007.01547.x.
7. Adams MR. Safety of industrial lactic acid bacteria. J Biotechnol 1999;68(2-3):171-8.
8. Besselink MG, van Santvoort HC, Buskens E, et al. Probiotic prophylaxis in predicted severe acute pancreatitis: a randomised, double-blind, placebo-controlled trial. Lancet 2008;371(9613):651-9. doi: 10.1016/S0140-6736(08)60207-X.
9. Besselink MG, van Santvoort HC, Renooij W, et al. Intestinal barrier dysfunction in a randomized trial of a specific probiotic composition in acute pancreatitis. Ann Surg 2009;250(5):712-9. doi: 10.1097/SLA.0b013e3181bce5bd.
10. van Baarlen P, Troost F, van der Meer C, et al. Human mucosal in vivo transcriptome responses to three lactobacilli indicate how probiotics may modulate human cellular pathways. Proc Natl Acad Sci U S A 2011;108 Suppl 1:4562-9. doi: 10.1073/pnas.1000079107.
11. Van Wijck K, Lenaerts K, Van Bijnen AA, et al. Aggravation of exercise-induced intestinal injury by Ibuprofen in athletes. Med Sci Sports Exerc 2012;44(12):2257-62. doi: 10.1249/MSS.0b013e318265dd3d.
12. Troost FJ, Saris WH, Brummer RJ. Recombinant human lactoferrin ingestion attenuates indomethacin-induced enteropathy in vivo in healthy volunteers. Eur J Clin Nutr 2003;57(12):1579-85. doi: 10.1038/sj.ejcn.1601727.
13. Bours MJ, Troost FJ, Brummer RJ, Bast A, Dagnelie PC. Local effect of adenosine 5'-triphosphate on indomethacin-induced permeability changes in the human small intestine. Eur J Gastroenterol Hepatol 2007;19(3):245-50. doi: 10.1097/MEG.0b013e328011093c.
14. de Jager W, te Velthuis H, Prakken BJ, Kuis W, Rijkers GT. Simultaneous detection of 15 human cytokines in a single sample of stimulated peripheral blood mononuclear cells. Clin Diagn Lab Immunol 2003;10(1):133-9.
15. Salonen A, Nikkila J, Jalanka-Tuovinen J, et al. Comparative analysis of fecal DNA extraction methods with phylogenetic microarray: effective recovery of bacterial and archaeal DNA using mechanical cell lysis. J Microbiol Methods 2010;81(2):127-34. doi: 10.1016/j.mimet.2010.02.007.
16. van den Bogert B, de Vos WM, Zoetendal EG, Kleerebezem M. Microarray analysis and barcoded pyrosequencing provide consistent microbial profiles depending on the source of human intestinal samples. Appl Environ Microbiol 2011;77(6):2071-80. doi: 10.1128/AEM.02477-10.
17. Leps J, Smilauer P. Multivariate analysis of ecological data using CANOCO. In: Press CU, ed. Cambridge, 2003.
